# The characteristics of depression and sleep among Chinese high school students: A cross-sectional study

**DOI:** 10.1097/MD.0000000000051117

**Published:** 2026-10-16

**Authors:** Herun Han, Ziwen Mu, Qiran Li, Hongtao Zeng, Yixuan Ma, Zhihua Liu, Chen Li, Yuxuan Zhou, Zhiguang Guo, Meng Lv, Xiao Zhou

**Affiliations:** a School of Physical Education of Huazhong University of Science and Technology, Wuhan, China; b School of Marxism, Huazhong University of Science and Technology, Wuhan, China; c Division of Sports Science and Physical Education, Tsinghua University, Beijing, China; d Wushu Administrative Center of General Administration of Sport of China, Beijing, China; e School of Physical Education of South-Central Minzu University, Wuhan, China; f School of Sports Health, HuBei University of Chinese Medicine, Wuhan, China; g HuaiBei No.12 High School, Huaibei, China.

**Keywords:** adolescent, depression, prevalence, sleep quality

## Abstract

Mental health and sleep problems are among the most significant burdens of illnesses for adolescents in China. This study aimed to investigate the characteristics of depression and sleep quality among high school students and compare sleep quality across students with varying levels of depression. A cross-sectional study was performed to collect data on depression and sleep quality using a paper questionnaire including a self-rating depression scale (SDS) and Pittsburgh Sleep Quality Index (PSQI). Among all the 537 high school students, 57.0% (306 participants) experienced depression and 47.5% (255 participants) suffered from poor sleep quality. Males showed significantly greater SDS scores than females (55.2 ± 7.2 vs 53.8 ± 6.8, *P* = .027). Significantly greater PSQI score in high school students with moderate-to-severe depression than those with mild depression and nondepression (5.2 ± 2.7 vs 5.3 ± 2.4 *vs* 6.2 ± 2.3, *F* = 3.5, *P* = .032). In contrast, an adjusted logistic regression model indicated that depression severity was not an independent significant predictor of poor sleep quality after adjusting for age, sex, and BMI (all *P* > .05). Overall, high school students exhibited a high prevalence of depression and poor sleep quality, with those experiencing moderate-to-severe depression showing the poorest sleep quality in unadjusted comparisons. However, depression severity did not remain an independent significant predictor of poor sleep quality after adjustment. Depression and sleep problems among high school students should receive greater attention, particularly for high school students with moderate-to-severe depression.

## 1. Introduction

Depression is a major mental health problem globally, and it not only affects the mental health of adolescents, but also has a negative impact on their quality of life.^[1]^ A national survey in Bangladesh found that the overall prevalence of depression among adolescents is 24.5%.^[2]^ In a nationally representative survey in the United States, the prevalence of major depression was found to be 2.3% among adolescents aged 13 to 18 years.^[3]^ In China, the prevalence of depression among adolescents is approximately 20.0% to 43.7%.^[4-7]^ Moreover, the prevalence of depression increased with grade level, with a gradual increase from the first year of middle school to the third year of high school.^[5]^ The positive rate of major depressive disorder (MDD) in adolescents was 2.0%, which was higher than in previous surveys.^[8]^ More serious is the fact that adolescents suffering from MDD have a higher tendency to commit suicide, at a rate of 38.2%.^[9]^ Approximately 9.3% to 16.1% of the group of adolescents have depression. According to data from the Seventh Population Census of the National Bureau of Statistics, in 2020, the population of young people in China aged between 10 and 19 is projected to be 158 million, accounting for 11.2% of the country’s population.^[10]^ If this ratio is taken into account, the number of adolescents suffering from depressive symptoms approximately range from 38.5 million to 66.7 million, or 2.7% to 4.7% of the country’s total population. The number of adolescents suffering from depression who tend to be suicidal may be approximately 3.6 million to 11 million.

A significant relationship was found between sleep quality and depressive symptoms, with different sleep qualities leading to different levels of depressive symptoms.^[11,12]^ Individuals with sleep disorders have a higher prevalence of depressive symptoms than those without.^[13]^ Moreover, poor sleep quality (such as sleep-onset insomnia and hypersomnia) and short sleep duration (<6 h/d) were significantly associated with higher depressive severity in adolescents.^[12,14]^ Past studies have shown that adolescents with poor sleep quality were 2 to 5 times more likely to suffer from depressive symptoms or even MDD than those with good sleep quality.^[15,16]^

In addition to depression, numerous studies on sleep have shown that sleep quality in adolescents and the prevalence of sleep disorders across countries are similar. For example, in Iran, 31.9 % of adolescents suffer from sleep disorders.^[17,18]^ In Norway, the prevalence of sleep disorders among adolescents is approximately 23.8%, and the severity of sleep disorders is higher among girls than boys.^[19]^ However, in China, a multi-center study found that more than two-thirds of adolescents over 11 years of age suffer from sleep disorders.^[20]^

For the physical and mental health of adolescents, it is important to pay attention not only to the prevalence of depression and the factors affecting it but also to the quality of sleep among adolescents. Nonetheless, studies on depression and sleep in Chinese adolescents are scarce. Therefore, this study aimed to investigate the prevalence of depressive symptoms and the distribution of sleep quality, and to test the hypothesis that sleep quality deteriorates progressively with increasing severity of depressive symptoms among high school students based on prior evidence.^[21]^

## 2. Materials and methods

### 2.1. Participants

A cross-sectional, questionnaire-based descriptive study was conducted in Huaibei City, Anhui Province, China, in 2023. A total of 619 high school students aged 15 to 18 years were recruited.

### 2.2. Self-reported questionnaire survey

A self-reported questionnaire included 3 sections: a demographic survey, Pittsburgh Sleep Quality Index (PSQI) questions, and a self-rating depression scale (SDS) was used to collect data. The first section is the demographic survey, including gender, age, height, weight, body mass index (BMI, calculated by height and weight values of the participants), and past medical history. The second section is the PSQI for sleep quality collection. The PSQI consists of 19 self-rated questions, with a total score ranging from 0 to 21, with higher scores indicating poorer sleep quality. It took subjects 5 to 10 minutes to complete the test questions. The 19 items were divided into 7 components: sleep quality, sleep latency, sleep duration, sleep efficiency, sleep disorders, use of sleep medication, and daytime dysfunction. Participants with PSQI scores above 5 were categorized as having poor sleep quality based on the criteria established by Buysse et al.^[22]^

The third section is the self-rating depression scale (SDS), which contains 20 items, 10 positive and 10 negative, each rated on a 4-point scale from 1 (none or very little) to 4 (most or all of the time), and assesses mood symptoms over the past 7 days.^[23]^ In this scale, there were 10 items describing positivity and scored oppositely. The raw scores were multiplied by 1.25 and then rounded to the nearest decimal point to convert to the final index score for SDS. The severity of depressive symptoms was categorized according to the index score as follows: no depression (index score < 50), mild depression (index score 50–60), moderate depression (index score 60–70), and severe depression (index score > 70).

A total of 619 participants were recruited prior to the study survey, and the inclusion criteria for the questionnaire were understanding of how to complete the entire scale and the meaning of each question, having completed the questionnaire and the informed consent form, and high school students aged 18 years and below. The exclusion criteria were not completing the questionnaire; having outliers or outliers in the values; alcohol abuse within 3 months; taking hormonal, depressive, and sleeping medications; and not completing the informed consent form. Based on the criteria of the International Classification of Diseases and the Diagnostic and Statistical Manual of Mental Disorders,^[24,25]^ the use of alcohol, hormones, or drugs may cause mental and behavioral disorders. Thus, we used this condition to screen the participants to address potential sources of bias. Ultimately, 537 participants met our inclusion criteria.

This study was reviewed and approved by the Institutional Ethics Board of Tongji Medical College, Huazhong University of Science and Technology, China (Notification Number [2023] IEC (S172)). This study adhered to the ethical principles of the Declaration of Helsinki for Human Research. All participants and guardians of the minors were required to provide written informed consent before participating in the study.

### 2.3. Statistical analysis

We used the Shapiro–Wilk test to check the normality of the demographic parameters (i.e., age, height, weight, and BMI) and points of the PSQI and SDS. The Mann–Whitney *U* test was used for statistical analysis of the demographic parameters (abnormal distribution) between the male and female participants. An independent sample *t* test was used for statistical analysis of the PSQI and SDS data presenting normal distribution. Furthermore, the participants were stratified into 3 groups based on the intensity of depression. One-way analysis of variance (ANOVA) followed by Tukey’s test was used for statistical analysis of the differences in PSQI scores among the 3 groups. Effect sizes (Cohen *d* for 2 groups and partial *η*^*2*^ for 3 groups) were calculated and were interpreted. In addition, logistic regression models were used to examine the association between depression and sleep quality. Model-adjusted variables included gender, age, height, weight, and BMI. Analyses were conducted using SPSS Statistics (IBM v.27, Armonk). A *P*-value less than .05 was set as the level of significance.

## 3. Results

A total of 537 adolescents, including 243 (45.3%) males and 294 (54.7%) females, met the inclusion criteria (Fig. 1). Table 1 shows the basic information of all participants broken down by gender. There were significant differences between the male and female students in terms of age (*P* = .02), height (*P* < .01), weight (*P* < .01), and BMI (*P *< .01).

**Table 1:** Demographic parameters of all participants by gender.

Variable	Total% (n = 537)	Male% (n = 243)	Female% (n = 294)	*P*
Age, yr	16.2 ± 0.5	16.3 ± 0.5	16.2 ± 0.5	.02
Height, cm	170.7 ± 8.1	177.5 ± 5.6	165.1 ± 4.9	.000
Weight, kg	63.6 ± 14.0	71.8 ± 14.4	56.7 ± 9.2	.000
BMI, kg/m^2^	21.7 ± 3.8	22.8 ± 4.3	20.8 ± 3.0	.000

Data are presented as mean ± standard deviation.

BMI = body mass index, *P*-value = between males and females.

**Figure 1.**
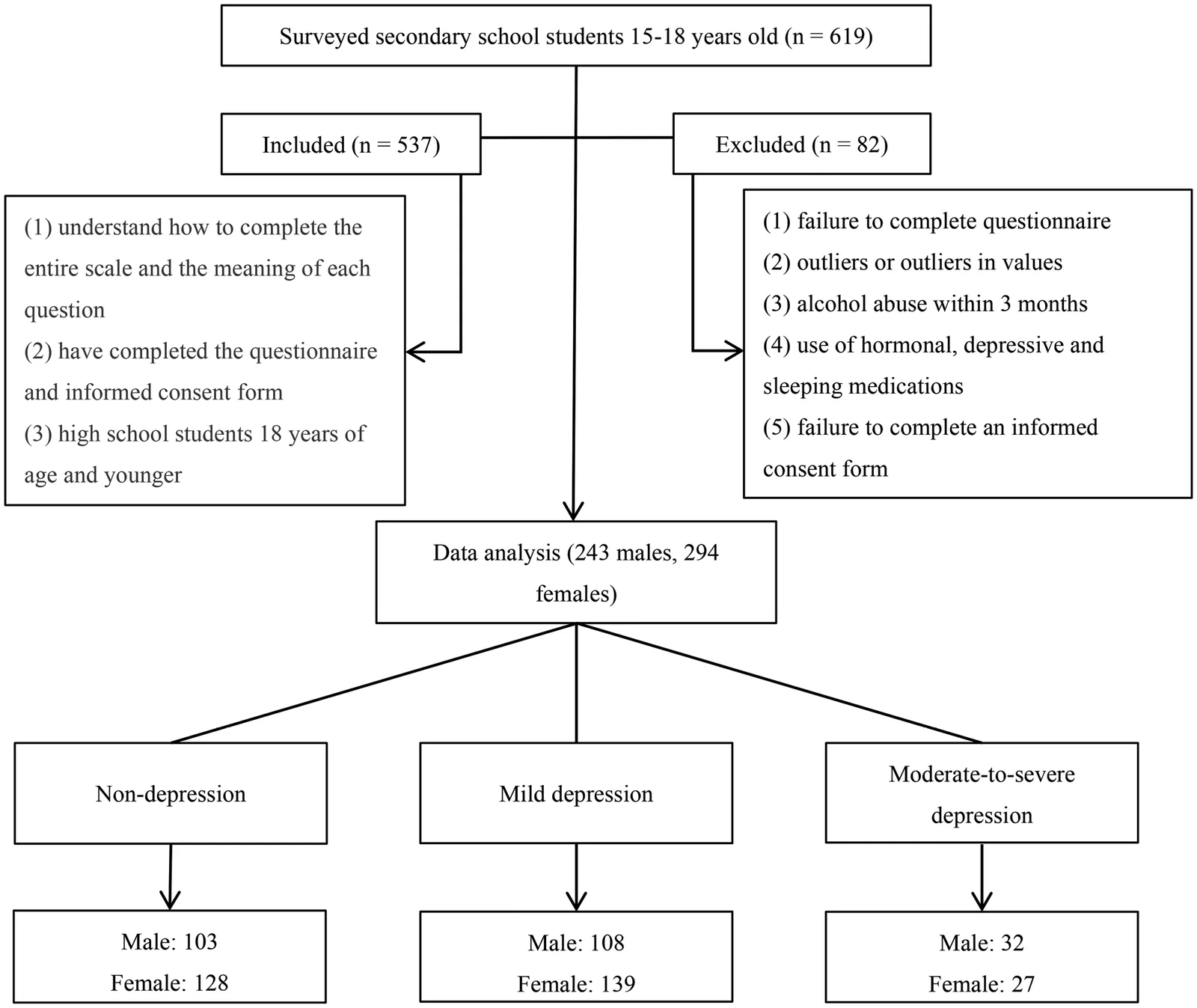
Screening flow.

Table 2 presents the scores for different depressive symptoms as well as scores for different sleep qualities. The mean SDS was 54.4 ± 7.0. Significant differences in the mean SDS score were found between the male and female students (males: 55.2 ± 7.2 vs females: 53.8 ± 6.8, *P* = .027). In terms of the distribution of different depressive symptoms, 306 out of 537 adolescents showed depressive symptoms, including 140 males and 166 females. There were 247 adolescents (108 males; 139 females) with mild depressive symptoms, 55 adolescents (29 males; 26 females) with moderate depressive symptoms, and 4 adolescents (3 males and 1 female) with severe depression.

**Table 2:** Comparison of SDS and PSQI measurements by sex.

Variable	Total (n = 537)	Male (n = 243)	Female (n = 294)	*P*
SDS	54.4 ± 7.0	55.2 ± 7.2*	53.8 ± 6.8	0.027
Nondepressed (103 males; 128 females)	48.5 ± 4.1	49.4 ± 3.5**	47.8 ± 4.4	0.004
Mild depression (108 males; 139 females)	57.1 ± 2.4	57.1 ± 2.4	57.1 ± 2.4	0.866
Moderate depression (29 males; 26 females)	64.5 ± 2.2	64.4 ± 2.0	64.6 ± 2.4	0.715
Major depressive (3 males; 1 females)	90.6 ± 9.0	92.5 ± 10.0	85.0	-
PSQI	5.4 ± 2.5	5.5 ± 2.5	5.3 ± 2.6	0.499
Good sleep (122 males; 160 females)	3.6 ± 1.3	3.6 ± 1.2	3.5 ± 1.4	0.739
Poor sleep (121 males; 134 females)	7.4 ± 1.9	7.4 ± 1.9	7.5 ± 1.9	0.706

Data are presented as mean ± standard deviation.

SDS = self-rating depression scale, PSQI = Pittsburgh Sleep Quality Index.

* *P* value < .05.

** *P* value < .05 between male and female.

With regard to the distribution of different sleep qualities, among all 537 adolescents, 282 adolescents (122 males; 160 females) showed good sleep, while 255 (121 males; 134 females) showed poor sleep.

Figure 2 shows the prevalence of different levels of depressive symptoms among the high school students. Overall, the prevalence of depression among high school students was 57.0% (SDS score ≥ 50). 46.0% of high school students (20.1% of male students and 25.9% of female students) suffered from mild depressive symptoms, 10.2% suffered from moderate depressive symptoms (5.4% of male students and 4.8% of female students), and 0.7% suffered from severe depressive symptoms.

**Figure 2.**
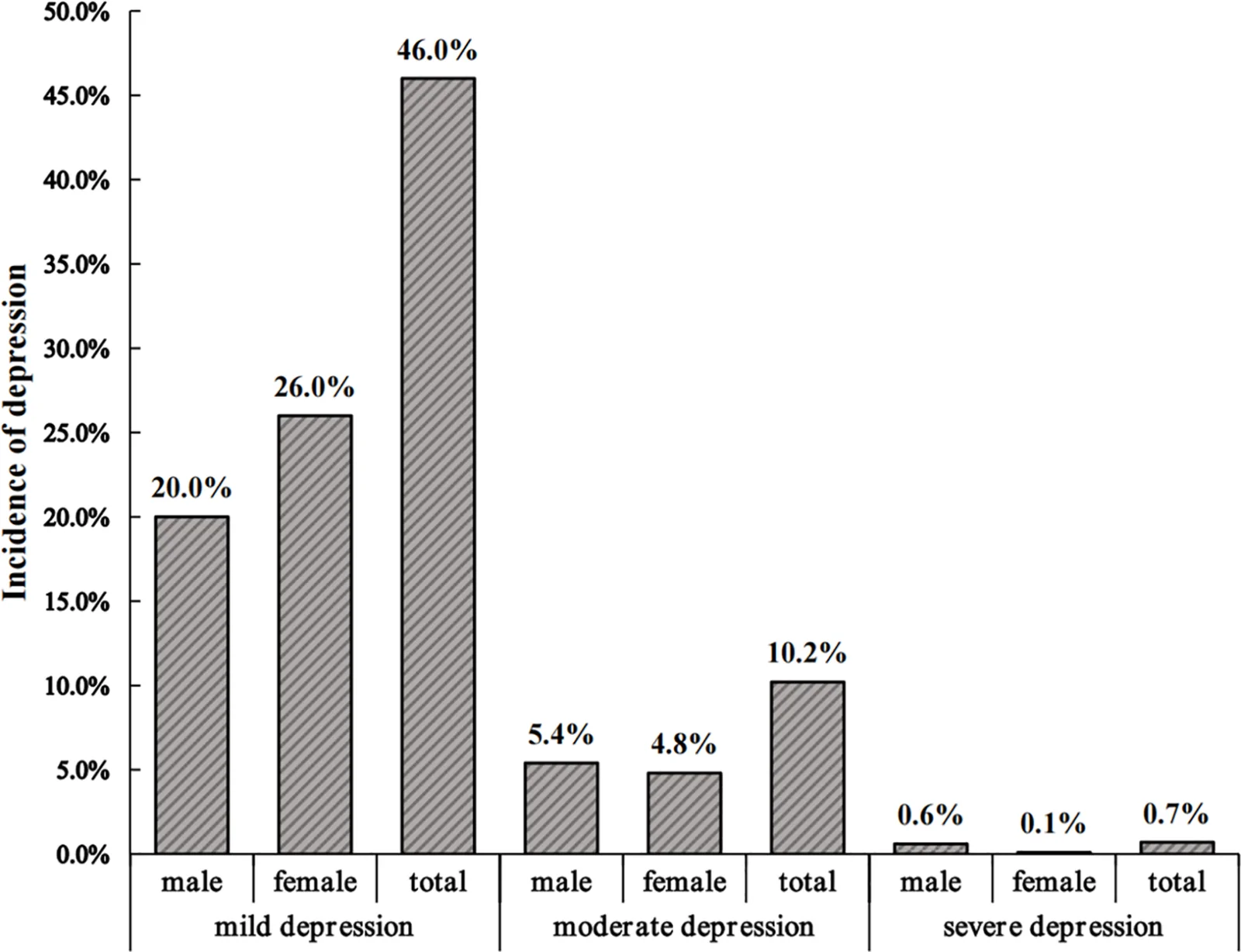
The prevalence of mild, moderate, and severe depression among high school students, broken down by gender.

For further analysis, the participants were divided into 3 groups (Table 3): nondepression, mild depression, and moderate-to-severe depression. A significant difference in sleep quality was observed between adolescents with no depressive symptoms, mild depressive symptoms, and moderate and severe depressive symptoms (*P* = .032, partial *η*^*2*^ = 0.013/small effect). Post hoc comparisons using Tukey’s HSD test revealed a statistically significant difference in sleep quality between adolescents with no depressive symptoms and those with moderate-to-severe depressive symptoms (5.2 ± 2.7 vs 6.2 ± 2.3, *P* = .029); however, the effect size was small (Cohen *d* = 0.381). In addition, a significant difference in sleep quality was found between adolescents with mild depressive symptoms and those with moderate-to-severe depressive symptoms (5.3 ± 2.4 vs 6.2 ± 2.3, *P* = .042), with a small effect size (Cohen *d* = 0.378).

**Table 3:** Comparison of sleep quality by different depression level.

Variable	Nondepression	Mild depression	Moderate to severe depression	*F*	*P*	Partial η^*2*^
PSQI	5.2 ± 2.7*	5.3 ± 2.4†	6.2 ± 2.3*^,^†	3.5	.032	0.013

Values are mean ± standard deviation.

PSQI = Pittsburgh Sleep Quality Index.

* *P *< .05, between adolescents with no depressive symptoms and those with moderate and severe depressive symptoms.

† *P *< .05, between adolescents with mild depressive symptoms and those with moderate to severe depressive symptoms.

To examine the independent association, a multivariable logistic regression was performed with poor sleep quality as the dependent variable and depression severity as the independent variable, adjusted for age, sex, height, weight, and BMI. No statistically significant association between depression severity and sleep quality across the crude model, model 1, and model 2 (all *P* > .05; Table 4).

**Table 4:** Logistic regression analysis of the association between sleep quality and depression severity.

Variable	Crude			Model 1			Model 2		
	OR	95% CI	*P*-value	OR	95% CI	*P*-value	OR	95% CI	*P*-value
Depression									
Nondepressed	1.00	–	–	1.00	–	–	1.00	–	–
Mild	1.02	0.70–1.48	.921	1.01	0.70–1.47	.951	1.00	0.69–1.47	.992
Moderate- to-severe	1.48	0.82–2.67	.193	1.45	0.80–2.62	.219	1.49	0.82–2.71	.191
Gender									
Male	–	–	–	1.00	–	–	1.00	–	–
Female	–	–	–	1.17	0.82–1.63	.408	0.67	0.40–1.16	.156
Age	–	–	–	1.10	0.77–1.58	.590	1.08	0.75–1.55	.683
Height	–	–	–	–	–	–	1.02	0.94–1.10	.718
Weight	–	–	–	–	–	–	1.03	0.93–1.14	.554
BMI	–	–	–	–	–	–	0.95	0.71–1.28	.735

BMI = body mass index, CI = confidence interval, OR = odds ratio.

## 4. Discussion

This study supports the notion that adolescent depression is prevalent in China, and over half of high school students experience depression. Almost half of all participants suffered from mild depression. Moreover, the prevalence of depression among male students was significantly higher than that among female students. Meanwhile, almost half of the high school students had poor sleep. Participants with mild or moderate-to-severe depression showed a higher score for poorer sleep quality than those without.

According to previous studies, mental health and depression among secondary school students aged 14 to 18 years (junior high school students: 58.5% of all participants; high school students: 41.5% of all participants) in Anhui province, China, showed that 20.0% of the students had depression problems.^[4]^ In another study on the prevalence of depression in students aged 7 to 17 years in central and western China, an overall prevalence of 23.9% risk of depression was found.^[6]^ The latest systematic and meta-analysis review, which grouped children and adolescents together, reported that the average prevalence of depression was 26.7%, ranging from 3.0%-73.2%; high school students demonstrated a higher prevalence of depression than students of other ages.^[26]^ In this study, high school students showed a high prevalence of depression, accounting for 57.0%, which is higher than in previous studies. The observed difference may be attributed to the wide age range in previous studies.

In addition, the prevalence of depression among American and Japanese high school students was 36.7% and 17.3%,^[27,28]^ respectively, which was lower compared with that of high school students in our study. Chinese students face tremendous academic pressure at all stages of schooling, especially at the high school level. According to previous studies, 80.6% of Chinese high school students reported that “the most important thing to do as a high school student is to study” which was significantly higher than in America (50.5%) and Japan (46.2%). The proportion of Chinese students who worried about their academic performance (78.4%) has been reported to be higher than in the other 2 countries (58.1% in Japan and 54.6% in America).^[29]^ This evidence may explain the high prevalence of depression among the participants in the current study. In East Asia, a degree from a prestigious university is often seen as the only way to climb the social ladder. This is especially true for Chinese students, who face greater academic expectations and pressure from their families. The toxicity of educational pressure in East Asia lies in the resonance effect of institutional competition and family cultural expectations: the education system narrows the value of life down to scores and rankings, while families become the grassroots enforcers of this system.^[30,31]^

A notable finding was that male students exhibited significantly higher SDS scores than females (44.1 ± 5.8 vs 43.0 ± 5.4, *P* = .027). This contrasts with the majority of global literature, which typically reports higher depression prevalence and symptom severity among adolescent females. While speculative, several culturally specific factors might contribute to this observation within our sample of Chinese high school students.^[4,32,33]^ Possible explanations for these differences include the following: Chinese male senior high school students experience a combination of various academic pressures at different stages of life, particularly during the final year before the National College Entrance Examination, along with the conflicts arising from adolescent rebelliousness;^[34]^ Most male high school students mature later than female students, potentially experiencing greater academic pressure in their last year of high school. This is supported by Angold et al who found that, in mid-adolescence, boys had a higher prevalence of depression than girls.^[35]^ However, it is important to interpret this gender difference with caution. Due to the limited sample size of the severely depressed group, our analyses did not test for interaction effects such as gender and depression severity. The use of self-report questionnaires might also influence gender-specific reporting patterns in this cultural context. This finding warrants replication in future studies with larger samples, incorporating clinical interviews and exploring potential cultural moderators of gender differences in depressive symptom expression among adolescents facing high academic pressure.

The results observed in participants with mild or moderate-to-severe depression indicated that higher sleep disturbance scores may stem from shared neurobiological pathways. Chronic stress, prevalent in this high-achieving student population, can dysregulate the hypothalamic-pituitary-adrenal (HPA) axis, a feature observed in a significant proportion of depressed patients.^[36,37]^ Given that HPA axis dysfunction is intricately linked to both mood dysregulation and sleep disturbances,^[37]^ this potential biological underpinning warrants further investigation in the context of adolescent academic stress.

In addition, our findings indicated a significant difference in depressive symptoms and sleep quality among high school students, with those experiencing mild or moderate-to-severe depression exhibiting poorer sleep quality than those without depression. Based on previous studies, sleep quality is one of the most important factors that influence depressive symptoms.^[38]^ Specifically, compared to adolescents without depression, adolescents diagnosed with depression reported significantly more sleep disturbances, including decreased sleep quality and symptoms of insomnia and hypersomnia.^[12]^ Studies on depressive symptoms and sleep quality in adolescents are scarce. A study of female adolescents showed significant positive correlations between PSQI scores, depression, and self-reported learning problems.^[39]^ In addition, a study on depressive symptoms and sleep quality among college students reported significant differences in the impact of the severity of depressive symptoms on sleep quality. Students with mild, moderate, or severe depressive symptoms exhibited poorer sleep quality than did those with no or minimal symptoms.^[40]^

In the adolescent population, the risk of depression was significantly increased in adolescents with poor sleep quality and shorter sleep duration, whereas good sleep quality significantly reduced the risk of depression.^[12]^ In our study, we focused on the prevalence of depressive symptoms and sleep quality among high school students, in addition to the differences between different levels of depressive symptoms and sleep quality. Our results showed significant differences in sleep quality across depressive symptoms. In particular, there was a significant difference in sleep quality between no depressive symptoms and moderate or severe depressive symptoms, and a significant difference in sleep quality between mild depressive symptoms and moderate or severe depressive symptoms. This finding suggests that the severity of depressive symptoms may directly affect sleep, which may be a significant predictor of depression. People with poor sleep quality are at a higher risk of developing depression.^[41]^

The relationship between sleep and depression involves multiple neurotransmitter systems, including serotonergic, cholinergic, and noradrenergic systems.^[42]^ Serotonin, in particular, plays a key regulatory role in mood, cognition, learning, memory, rewards, and sleep.^[42]^ In women, hormonal fluctuations during physiological stages, such as the menstrual cycle, can affect serotonin levels, thereby increasing the risk of depression and disrupting sleep. In contrast, men generally maintain more stable serotonin levels, but chronic stress and changes in testosterone levels can reduce serotonin levels, potentially disrupting sleep.^[42,43]^ Furthermore, sleep disorders exacerbate neurodegeneration, whereas neurodegenerative diseases disrupt sleep, creating a vicious cycle of depression and sleep disturbances.^[44]^ In other words, sleep disorders significantly contribute to the onset and severity of depression in adolescents, and depression itself often leads to sleep disruption, creating a vicious cycle that exacerbates both of these awful conditions.^[45]^ These findings support the results of our study, which explains why participants with mild or moderate to severe depression showed poorer sleep quality than those without.

Depression has several contributing factors, and previous research has suggested that it may be linked to abnormal functioning in specific areas of the brain.^[46]^ For the adolescent population, regular breakfast consumption of green and yellow vegetables appears to have a significant impact on adolescent mental health and is associated with a reduction in depressive symptoms.^[47]^ Additionally, in a 12-week controlled trial of music therapy, a significant difference in depression scores was found between the group that used music therapy techniques and the group that did not use music therapy techniques.^[48]^

Physical activity has a positive effect on depression and sleep quality in adolescents.^[49,50]^ Moreover, Chinese students spend too much time on sedentary activities (a risk factor for sleep disorders) during their leisure time.^[51]^ Moderate-intensity physical activity is a better option for adolescents with depression and poor sleep quality.^[49,50]^

Notably, the present study observed a significant association between depression severity and poorer sleep quality in the unadjusted, continuous-outcome analysis, yet the adjusted categorical logistic regression did not identify depression severity as an independent significant predictor of poor sleep quality. Several considerations may explain this apparent discrepancy. First, dichotomizing the continuous PSQI into “poor” versus “good” sleep reduces statistical power and discards information, attenuating a weak association below the significance threshold; the ANOVA retained the full continuous scale. Second, the effect sizes in this sample were uniformly small (Cohen *d* of approximately 0.38; partial eta squared = 0.013), so the absolute sleep-quality difference across depression-severity groups was modest and more readily detected by the larger effective sample of the continuous ANOVA than by the dichotomized logistic model. Third, the moderate-to-severe subgroup was relatively small (n = 59, 11.0% of the sample, including only 4 severe cases), yielding wide confidence intervals that limit the precision of the adjusted odds ratios. Fourth, within this uniformly high-pressure student population – where the prevalence of depression (57.0%) and poor sleep (47.5%) were both elevated – the overall distribution of sleep quality was shifted upward, potentially creating a ceiling effect that constrains the incremental explanatory power of depression severity. Finally, shared upstream determinants such as academic burnout and anxiety may jointly influence both depression and sleep quality,^[52]^ attenuating the independent contribution of depression severity in the adjusted model. Collectively, the absence of an independent association in the adjusted model is best interpreted as indicating a weak and non-robust relationship rather than no relationship.

Some limitations in this study should be acknowledged. First, self-report questionnaires (SDS and PSQI) may cause reporting biases, such as social desirability bias or difficulties in accurate self-assessment, which are recognized challenges in adolescent mental health research.^[53]^ Future study could enhance the data collection quality by supplementing self-report data with objective measurement methods, such as activity profiles for sleep assessment and structured clinical interviews for depression diagnosis. Second, it is crucial to emphasize that this study utilized a cross-sectional design. Third, we observed poorer sleep quality among students with moderate-to-severe depression compared to those with mild or no symptoms. However, the association between depression and sleep quality was not found using logistic regression models, which might be due to the small size of moderate and severe depressive participants. Additionally, in the context of Chinese education, the factors influencing high school students’ depression and sleep quality are more complex, with academic burnout and anxiety, which also potentially impact their sleep quality,^[52]^ possibly accounting for the lack of association between depression and sleep quality among the participants in this study.

Despite these limitations, our findings highlight feasible targets for public health interventions and may inform school-based mental health interventions or sleep hygiene programs. To address the high prevalence of depression and poor sleep quality among Chinese high school students, particularly the significant increase in depression scores among males and the markedly poorer sleep quality in the moderate-to-severe depression group, we advocate for annual SDS/PSQI screening and prioritized intervention for high-risk subgroups,^[54]^ as well as the development of gender-sensitive counseling tailored to the unique stressors faced by male students in high-pressure academic environments.^[55]^

## 5. Conclusion

More than half of high school students experienced depression, and almost half had poor sleep quality, with students experiencing moderate-to-severe depression exhibiting the poorest sleep quality in unadjusted comparisons. Male students showed significantly higher SDS scores than female students. However, given the cross-sectional design and the absence of an independent association in the adjusted model, the depression-sleep quality link should be interpreted with caution. Depression and sleep problems among high school students warrant greater attention, particularly for those with moderate-to-severe symptoms.

## Author contributions

**Conceptualization:** Meng Lv, Xiao Zhou.

**Data curation:** Hongtao Zeng, Chen Li, Zhiguang Guo, Xiao Zhou.

**Formal analysis:** Herun Han, Ziwen Mu, Qiran Li, Zhihua Liu.

**Funding acquisition:** Yuxuan Zhou, Xiao Zhou.

**Writing – original draft:** Herun Han, Ziwen Mu.

**Writing – review & editing:** Hongtao Zeng, Yixuan Ma, Yuxuan Zhou, Xiao Zhou.
